# Infinite Generation of Language Unreachable From a Stepwise Approach

**DOI:** 10.3389/fpsyg.2019.00425

**Published:** 2019-03-19

**Authors:** M. A. C. Huybregts

**Affiliations:** Utrecht Institute of Linguistics OTS (UIL-OTS), Utrecht University, Utrecht, Netherlands

**Keywords:** evolution, communicative pressures, infinite generation, productivity, computable/decidable, recursive, competence, strong/weak generation

## Abstract

Language is commonly thought of as a culturally evolved system of communication rather than a computational system for generating linguistic objects that express thought. Furthermore, language is commonly argued to have gradually evolved from finite proto-language which eventually developed into infinite language of modern humans. Both ideas are typically integrated in accounts that attempt to explain gradual evolution of more complex language from the increasingly strong pressures of communicative needs. Recently some arguments have been presented that the probability of the emergence of infinitely productive languages is increased by communicative pressures. These arguments fail. The question whether decidable languages evolve into infinite language is vacuous since infinite generation is the null hypothesis for a generative procedure. The argument that increasing cardinality leads to infinite language is incoherent since it essentially conflates concepts of performance with notions of competence. Recursive characterization of infinite language is perfectly consistent with finite output. Further, the discussion completely ignores a basic insight that language is not about decidability of weakly generated strings but rather about properties of strongly generated structures. Finally, the plausibility proof that infinite productivity evolves from finite language is false because it confuses (infinite) cardinal numbers with (natural) ordinal numbers. Infinite generation cannot be reached with a stepwise approach.

## Introduction

Language is commonly thought of as a culturally evolved system of communication (Dunbar, 1996, 2017; Tomasello, 2003, 2008; Kirby et al., 2007; Smith and Kirby, 2008; Chater and Christiansen, 2010; Kirby, 2017) rather than a computational system for generating linguistic objects expressive of thought (Chomsky, 2010, 2013, 2017a,b). Furthermore, it is also commonly argued that language has gradually evolved from finite proto-language which eventually developed into infinite language of modern humans (Bickerton, 1990; Pinker and Bloom, 1990; Corballis, 2002; Fitch, 2010; Jackendoff, 2010; Dediu and Levinson, 2013; Christiansen and Chater, 2015; Jackendoff and Wittenberg, 2017). Both ideas are typically integrated in accounts that attempt to explain gradual evolution of more complex language from the increasingly strong pressures of communicative needs (e.g., Smith and Kirby, 2008; Christiansen and Chater, 2015). In a recent article Piantadosi and Fedorenko (2017), P&F hereinafter, argue that “increasing the number of signals in a language increases the probability of languages that have—in fact—infinite cardinality. Thus, across evolutionary time, the productivity of human language could have arisen solely from algorithmic randomness combined with a communicative pressure for a large number of signals.” Here we will argue that these arguments fail. The question whether decidable languages evolve into infinite language is close to being vacuous since infinite productivity is the null hypothesis for a generative procedure. Decidable sets are equivalent to effectively computable functions (Gödel, 1934), which by default are characterized by infinite productivity and are “obtained in the ideal case where all of the practical restrictions on running time and memory space are removed” (Enderton, 1977, p. 530). Since infinite productivity is the null hypothesis, no communicative pressures are required for an internal system to emerge that conforms to laws of nature and third factor principles. Next, the argument that increasing cardinality leads to infinite language is incoherent since it essentially conflates concepts of *performance* with notions of *competence*. Recursive characterization of infinite language is perfectly consistent with finite output. Further, the discussion completely ignores a basic insight that language is not about decidability of *weakly generated* strings but rather about properties of *strongly generated* structures. Finally, the plausibility proof that infinite productivity evolves from finite language is false because it confuses (infinite) *cardinal* numbers with (natural) *ordinal* numbers. Infinite generation cannot be reached with a stepwise approach.

## Infinite Productivity of Computable Functions

The creative aspect of normal language use has been a nearly constant topic of philosophical/scientific discussion at least since Cartesian times (Descartes, Port-Royal grammarians), and even before (Galileo). Some qualifications are in order, e.g., last century's structural linguistics, grounded in behaviorist thinking, and recent preoccupation with item- or usage-based learning (Tomasello, 2003, 2008; Culicover and Jackendoff, 2005; Goldberg, 2006; Christiansen and Chater, 2015; Kirby, 2017), grounded in statistics-based domain-general learning from particulars. While the creative use of language is still mysterious, there has been substantial progress in understanding the principles underlying the unboundedness of human language (Chomsky, 2013).

Infinite productivity is the default property of a *computable function* (Turing, 1936; Davis, 1958; Watumull, 2012; Watumull et al., 2014). Consequently, if internal language is a computable function, a generative procedure defined in intension, there cannot be any non-arbitrary limit on its productivity. Imposing a limit requires an arbitrary stipulation, a complication that need be explained. Such explanation is provided by Yang (2016), who gives an explicit and enlightening account of how linguistic productivity is *determined* by his *Tolerance Principle* for arbitrary exceptions in specific domains of language. The alternative, rejecting language as a generative procedure leaves no question to investigate. Not only don't we know in that case what language is but absent any characterization of the set of well-formed sentences, P&F's question whether infinite productivity could arise from chance under communicative pressure becomes a mission impossible.

To make this perfectly clear, take one of the simplest formal languages imaginable, *L* = {*x* | *x* ϵ {*a*}^*^}, the language consisting of all strings on {*a*}. *L* contains an infinite number of strings, sequences of the symbol *a*, which could be recursively generated by a FS grammar *G(L)* with two rules only: (i) S → *a* S, and (ii) S → *a*. These rules generate *a, aa, aaa*, etc. A “simpler” finite language *L'* consisting of, say, five strings only, *L'* = {*a, aa, aaa, aaaa, aaaaa*}, can be characterized only by a more complex grammar *G(L')*, which has no less than nine rules.

(1) S → a
(2) S → a S1,   (3) S1 → a
(4) S → a S2,   (5) S2 → a S1
(6) S → a S3,   (7) S3 → a S2
(8) S → a S4,   (9) S4 → a S3

Grammar *G(L')* generates all five strings of *L'* and only these, permitting a maximum embedding depth of four. Here complexity of a grammar *G(L)* for any language *L* is understood as the length *l(G)* of the shortest description representing grammar *G* as measured by the number of rules relative to the cardinality of language *L*: the closest is *l(G)* to the number of signals, the more complex the language is. Evidently, finite generation is vastly more complex than generation of an infinite set. Infinite generation is therefore the null hypothesis. True, for finite languages, providing a finite list (or a set of rules that generate one terminal string each) may be simpler than any grammar generating the list's sentences. But in the example above even this finite list is more complex than the recursive grammar generating the infinite language with the arbitrary limit removed. For natural language the situation turns out to be dramatically more complex. Its generative grammar, a computable function, must therefore be recursive by default. Consequently, finite generation with its vast complexity could not have been an earlier step in evolution of language, precursing the simplicity of infinite generation.

There is no longest sentence in human language, which as a consequence must be infinite in size. Therefore, generating the unbounded array of structures requires a finitely specified recursive procedure. Now P&F “consider only languages *L* that are decidable,” but since decidable sets are equivalent to computable functions (Gödel, 1934), the latter ideally characterized by infinite productivity, infinity must be the null hypothesis for decidable sets as well. The conclusion must be that computable functions do not evolve into recursive systems with infinite productivity, because by default they start out being infinitely productive. Since human language is infinite and infinite generation is the null hypothesis, any limit on recursive application would be arbitrary, requiring an explanation. No evolution is required, therefore, and, in fact, as discussed in section Infinite Cardinal Numbers as Unattainable Limits, the idea of a stepwise approach to infinity is incoherent. P&F already presuppose what they set out to prove. The question of gradual evolution as a result of external pressures simply does not arise.

Still, this line of reasoning is not unique but shared along a broad spectrum of cognitive scientists (e.g., Scott-Phillips and Kirby, 2010; Christiansen and Chater, 2015; Kirby, 2017). These three studies are further misguided by hidden assumptions. Successful iterated-learning from finite collections of items actually happens in modern minds already fully equipped with recursive language/compositional meaning. Consequently, in these experiments “cultural evolution” does not contribute anything that wasn't already there before (Berwick and Chomsky, 2017). Rather these arguments presuppose what they conclude. Capacity for infinite generation need not be learned. It is something humans start out with in life. A recursive system has evolved, clearly, but its evolution must have been saltational, perhaps as a result of cerebral reorganization of an expanding brain. Language is therefore a biological isolate, a novelty, and emerged suddenly, recently in evolutionary time, and as far as we know with little or no evolutionary change since (Chomsky, 2010, 2017a,b; Berwick and Chomsky, 2011, 2016, 2017; Tattersall, 2012, 2017; Huybregts, 2017). In particular, no group differences have been found since we left Africa, and every child, African or non-African, can grow and develop any language of the community she is born into.

There is some intriguing evidence from computational simulation that suggests that nature would select the simplest stable recursive functions, the ones that produce the successor function (Minsky, 1985). And precisely this may have happened in the evolution of human language, which essentially is such a recursive function. No communicative pressures are needed to explain the urge to merge in language. Language's computational optimality may justify a bolder but no less cogent or plausible conjecture that “the basic principles of language may be drawn from the domain of (virtual) conceptual necessity” (Chomsky and Watumull, in a book chapter currently in press). Universal grammar would then be inevitably universal for both humans and aliens: absent any external selective pressures on an internal system, nature would converge on the same class of computational procedures that conform essentially to laws of nature and third factor principles only [Roberts, Watumull, and Chomsky, presentation “Universal Grammar” at the International Space Development Conference (ISDC), Los Angeles, 2018].

## Infinite Productivity and Finite Output

**Figure 1:**
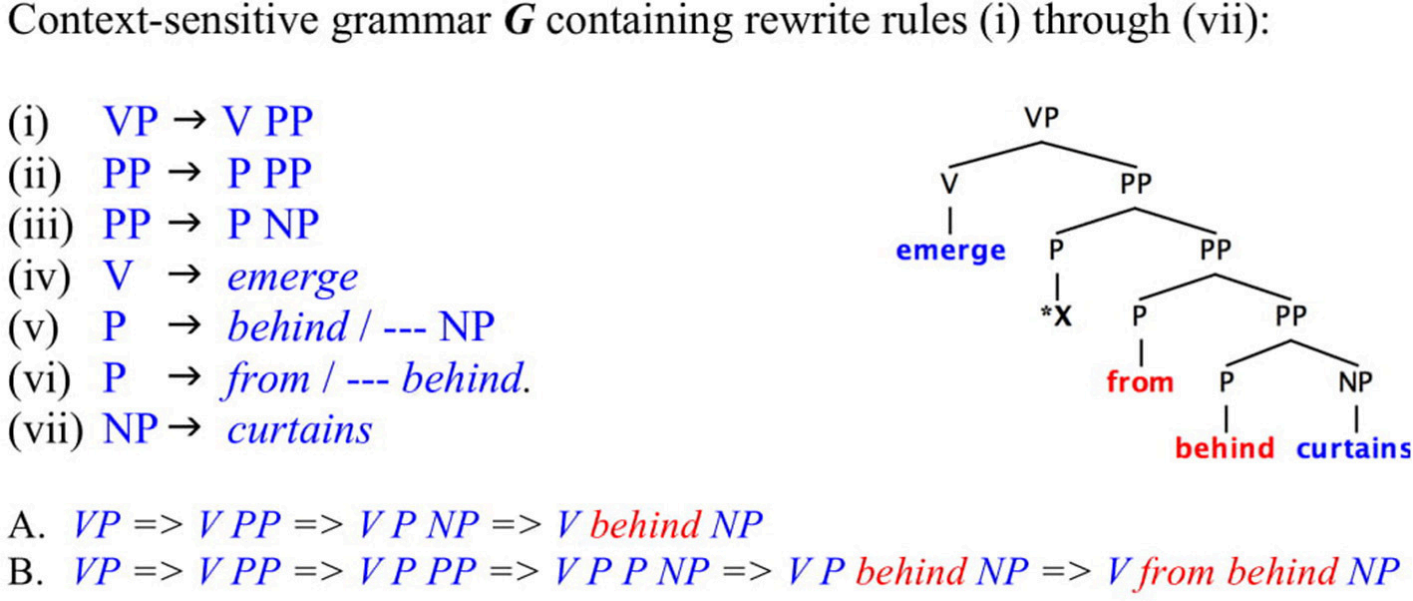
Recursive grammar but finite output. *CS* Phrase Structure Grammar ***G*** runs a recursive program that generates an indefinitely large number of nested prepositional phrases. Still its output is idiosyncratically restricted by context-sensitive rules that allow production of a finite set of linguistic objects only. Rule (ii) applies recursively but ***G'***s output is strictly finite, (A) *[emerge [behind curtains]]* and (B) *[emerge [from [behind curtains]]]*, due to the context-sensitive restrictions of rules (v) and (vi). These must be considered arbitrary lexical constraints relative to the freely applying recursive rule (ii). The phrase marker corresponding to the tree in this figure cannot terminate in a well-formed verb phrase.

We should be careful, however, not to confuse the *infinite productivity* of the generative procedure (a function in intension) with the *cardinality* of the decidable set of structures it generates (the well-formed formulas defined in extension). To recursively characterize a set, applying an intensional function, is not to produce the set, represented as its output. Watumull et al. (2014) offers a principled discussion of these aspects of recursion theory. Without contradiction, finite outputs can be generated by recursive systems. See Figure 1 for an illustration of this.

Within the SMT framework of current generative theories (Chomsky, 2000, 2015, 2017c), a more empirically informed case may be the morpho-syntactic constraints on case stacking in Kayardild syntax that effectively restrict the output of the recursive operation to a single embedding at most (Evans and Levinson, 2009). The morpho-syntactic conditions restraining recursive embedding of possessive noun phrases in German or Dutch constitute another cogent and convincing case. There are two strategies for handling these possessive constructions in standard Dutch, a fully productive strategy that involves possessive agreement between possessor and possessee, and a partially productive one that involves genitive case agreement. The latter is more archaic and restricted to proper names, kinship terms and a few “genitive fossils” in the language. The discussion below is simplified somewhat for convenience (phrases (1a)/(2a) mean “the man's aunt,” and phrases (1b)/(2b) mean “the man's aunt's bike”).


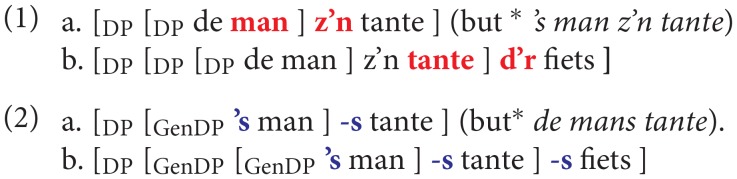


Modulo lexical restrictions on genitive case, each construction permits free recursion. Both structures are characterized by agreement between determiner and determiner phrase, Agree(D, DP): person/number/gender agreement in (1) and Case agreement in (2).

In (2) the case-marked head (recursively) agrees in Case with the case-marked determiner phrase in its specifier position. I.e., genitive case-marker “-s” (cliticized to the noun *man*) agrees with genitive determiner '*s* (contracted form of *des*) in (2a), and there is recursive agreement in (2b), where genitive “-s” of *tantes* agrees with genitive determiner phrase '*s mans*. In (1) possessive agreement features of the determiner recursively agree with its determiner phrase in person, number and gender: *z'n* ~ *de man* and *d'r* ~ *de man z'n tante*.

Interestingly, hybridization leads to a curious asymmetry of recursion, explained on present assumptions.


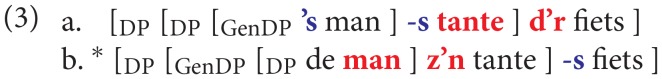


Free recursion is constrained here by an extraneous morphosyntactic factor: genitive case agreement cannot apply (but must apply) in (3b), whereas possessor-agreement must apply (and can apply) in (3a). Both the asymmetry and the constraint (“criterial agreement”) on recursive output receive a principled explanation.

While the limiting conditions on recursive output may receive independent and principled explanations, in the Dutch case labeling requirements on exocentric constructions (Chomsky, 2013, 2015), it is important to stress that these are independent of the recursive computation, and are extraneous to it. The widely publicized Pirahã case (Everett, 2005; Nevins et al., 2009) is no different. The basic operation Merge is part of the faculty of language, the human genetic endowment for language, and applies without exception in humans anywhere, in every language, and in every construction. Recursiveness is a property of the generative procedure applicable to any input, not a feature of its output, which may be arbitrarily constrained by complicating idiosyncratic factors independent of the procedure. The procedure may generate an infinite language but only produce a finite subset of it.

Beyond the cases discussed above, there are other conclusive arguments for distinguishing a recursive generative procedure from its (in)finite output. Humans have I-language, a biological system with a computational capacity for generating a discrete infinity of hierarchical structures (“competence”), but their linguistic output (“performance”) must necessarily be strictly finite for other, biophysical reasons, quite extraneous to the generative procedure itself. Evidently, “unboundedness is a property of generative competence, not its application in performance” (Watumull et al., 2014).

Unfortunately, P&F do not stand alone in failing to distinguish these significant concepts. E.g., Christiansen and Chater (2015) recently argued for an account of the human ability to process “a limited amount of recursive structure in language” that derives from domain-general constraints on sequence learning, and “does not rely on recursion as a property of grammar.” According to Christiansen and Chater, “[T]he ability does not necessitate the postulation of recursive mechanisms to process them.” However, their account, building on construction grammar and usage-based approaches to language, leaves the problems unresolved. How are “constructions” and “limited recursive structure” characterized? Recursive characterization is ruled out by them apodictically. It is argued that “language is shaped by the brain” and but there is no answer to the question of why, how or when the brain did evolve a capacity to learn precisely those languages that are UG-compliant. As a result, none of these questions receive a decent answer. It is just not enough to say that what is observable is real (performance), and it is incorrect to assume that what is invisible is unreal (competence). It is the task of the sciences to reduce “complex visibles” to “simple invisibles” (Jean Baptiste Perrin, Nobel laureate in physics, 1926). Clearly, the distinction between the properties of a system and the use of that system cannot be in doubt. *Pace* empiricist prevalence, from finite output alone *nothing* can be inferred for internal language.

The suggestion that a theory that successfully ignores the distinction between competence (internalized system) and performance (externalized use of the system) should be the null-hypothesis (Christiansen and Chater, 2015; Piantadosi and Fedorenko, 2017) has the story backwards. The notion of an error of performance only makes sense by virtue of the existence of competence. How else could anything be an “error”? Further, there are double dissociations. There are competence effects that have no performative explanation, e.g., structure dependence of rules of grammar (Berwick et al., 2011). On the other hand, there are performative effects that cannot be explained by competence (self-embedding, garden path, blindspots). There may be peripheral impairments that preclude performance but leave competence intact. There are additional asymmetries in performance such as “agreement attraction errors” (Bock et al., 2001, and others). E.g., performance errors like *The key to the cabinets are on the table* occur significantly more frequently than attraction errors like *The keys to the cabinet is available*. To account for these skewed “attraction errors” we need an adequate theory of performance (probability) that presupposes competence (degrees of grammaticalness). Distinguishing between competence and performance is the null hypothesis. Deviation from it needs an argument and a “proof” of explanatory superiority.

## Strongly Generating the Language Phenotype

The *triple-I* qualification of language (*internal, individual, intensional*) highlights the elementary distinction between a generative procedure (“competence”) and its output production (“performance”). Language is *internal* to the *individual* in whose brain it is represented as a function in *intension*, a generative program (Chomsky, 1986). As a result, since recursion is a property of the computational procedure, not a characteristic feature of the program's output, it follows, in particular, that from a set of well-formed formulas nothing can be concluded about the nature of the generative procedure that generates them. Further, the notion of decidable sets relates to properties of their *weak generation*, the finite procedure that decides for each input if it belongs (“is grammatical”) or does not belong (“is ungrammatical”) to the set. But for the study of *natural* (as opposed to *formal*) language, *decidability* of sets, *weak generative capacity* (WGC), is not even a relevant notion, in sharp contrast with the generative procedure and its *strong generative capacity* (SGC), which is fundamental (Chomsky, 1956, 1965, 2007; Berwick, 1984). Despite much confused parlance otherwise, decidability of grammaticalness, relevant for formal language, is hardly an issue in the study of natural language, for which no surface structure characterization can be given.

For natural language, characterization of its unbounded array of hierarchical structures and their properties is fundamental, and proceeds from a finitary generative procedure. We can exemplify these matters, discussing some properties of the basic recursive operation Merge, defined as *Merge(X,Y)* = *{X,Y}*, where *X,Y* is a lexical item or a phrase *Z* that is itself the result of *Merge(X', Y')*. Binary *Merge(X,Y)* is thus an operation that takes two elements *X* and *Y* — *X,Y* atomic elements *or results of a previous Merge application* — and constructs a set out of them that contains just these elements as its members, imposing no further structure or ordering arrangement on them (Chomsky, 2015; Everaert et al., 2015). Since recursion is strictly the property of a rule to reapply to its own output, free Merge re-applying to its own output is recursive in this sense. Moreover, Merge must be allowed to operate on multiple syntactic objects in the workspace, applying in parallel to generate, e.g., both the syntactic objects *{this, man}* and *{loves, {that, woman}}* before merging these to construct a new syntactic object *{{this, man}, {loves, {that, woman}}}*. Consequently, *freely* applying Merge, defined by *induction*, **μ*****(x, y)*** = ***{x***, **μ*****(x', y')}***, will be *strongly generative* of structure, allowing for “unbounded expansion of the strongly generated structure.” Furthermore, “by these necessary and sufficient criteria the grammars of all natural languages are recursive” (Watumull et al., 2014). E.g., the structure externalized as “this man loves that woman” is defined by induction and strongly generated by recursive merge applications that cyclically build increasingly complex hierarchical structure: **μ*(*μ*(this*,** ***man)***, μ***(loves***, **μ*****(that, woman)))*** **=*GP* = >** ***{{this, man}, {loves, {that, woman}}}***.

For the study of language, not *grammaticality*, a property of *weak generative* adequacy, but rather *degree of grammaticalness*, a property of *strong generative* adequacy, is the linguistically significant concept (Chomsky, 1956, 1965). Formal languages exist by virtue of an *extensional* definition, and *any computational procedure* generating the language will suffice. It is only a *matter of choice*, not an empirical issue. In contrast, natural language cannot be characterized extensionally and are defined only *intensionally*. There is no choice of grammar (I-language) here: only one specific grammar must be selected since biology requires a single specific generative procedure, thus posing an empirical problem.

What is relevant is the nature of the intensional function that generates a discrete infinity of hierarchically structured expressions, a trait of the language phenotype, not the cardinality of the decidable set it generates. We want to know e.g., why the sentence *John knows a sharper lawyer than Bill* is ambiguous (i.e., between … *than Bill does* and … *than Bill is*), but why the sentence *John knows a lawyer who is sharper than Bill* shows constrained ambiguity (i.e., only … *than Bill is* could be a well-formed continuation). Note that underlying structures *John knows a sharp lawyer* and *John knows a lawyer who is sharp* are semantically equivalent. Therefore, disambiguation has no semantic, pragmatic or communicative causes but must result from properties of the computable program generating their syntactic structures (Everaert et al., 2015).

In fact, comparative constructions are analyzable as a special case of (unbounded) wh-movement of the compared element, and are constrained by conditions on movement (Chomsky, 1977). Examples like *John is richer than (what) you might expect him to be* or *You look much taller than (what) you really are* illustrate this movement theory of comparative deletion. Turning now to the ill-formed sentence, ^*^John knows a lawyer who is sharper than Bill does, we can see that ellipsis has applied to a VP, viz. *[*_*VP*_ *know a lawyer who is ec]*, which already contains a Complex NP Island violation (Chomsky, 1977). The derivation does not converge and non-convergence is explained by restrictions on computational resources, e.g., a phase impenetrability condition on merge-generated phrase structure (Chomsky, 2000). Complex analysis of hierarchical structure (strong generative capacity), not information about class membership (weak generative capacity), provides us with a clear understanding of why ambiguity is constrained, unifying comparative deletion with unbounded movement. Significantly, we do not content ourselves with simply observing that expressions are (un)acceptable. Even if there would be an observationally adequate approximation of acceptability there is no answer as to why they are (un)acceptable. Analogously, we want to know why the stars shine, not merely observe that they shine (Feynman, 1963/2011).

In natural language, a well-defined *Merge* operation strongly generates a digitally infinite array of hierarchically structured expressions that each receive systematic and determinate interpretations at the cognitive and sensorimotor systems interfacing with internal language. The interface mappings show asymmetry, with the mapping to the thought systems being primary and externalization at the sensorimotor systems ancillary (Everaert et al., 2015, 2017; Huybregts et al., 2016; Chomsky, 2017a,b). Precise insight into the nature of the generative procedure, the properties of the hierarchical structures it generates (Chomsky, 1965, 1981, 2007, 2015, 2017c; Everaert et al., 2015), its growth and development in the language learning child (Crain and Thornton, 2011; Crain, 2012; Piattelli-Palmarini and Berwick, 2012; Yang et al., 2017), development and representation in the human brain (Musso et al., 2003; Moro, 2016; Friederici et al., 2017) as well as its evolutionary origin (Tattersall, 2012, 2017; Berwick and Chomsky, 2016, 2017; Huybregts, 2017) are the primary goals of generative linguistic research, not class membership of individual sentences.

Study of evolution of the language faculty (UG) should therefore be primarily concerned with the emergence of a specific generative procedure in evolutionary time, and within a narrow evolutionary channel defined by historical contingencies and biophysical constraints (Monod, 1972), not with the probability of an infinitely productive language arising “solely from algorithmic randomness combined with a communicative pressure for a large number of signals.” Communicative efficiency does not govern language though computational efficiency does, unambiguously, ubiquitously, and unexceptionally (Chomsky, 2010, 2013, 2017a,b; Everaert et al., 2015). Algorithmic randomness begs the question of the prior availability of algorithms, computable functions with an inherently infinite productivity. The assertion that infinite human language may have arisen “solely” from these two factors only exarcerbates the incongruence and incoherence of the proposal. Evidently, UG is a *species-specific* and *domain-specific* biological system that did not evolve from algorithmic randomness or communicative pressure. For better informed accounts of why language came to stick with only us see (e.g., Tattersall, 2012, 2017; Bolhuis et al., 2014; Hauser et al., 2014; Berwick and Chomsky, 2016, 2017; Chomsky, 2017a,b).

Other linguists, e.g., Bickerton (1990, 2000), Jackendoff (1999, 2002), Jackendoff and Wittenberg (2017), Progovac (2009, 2016) have argued for a proto-language precursor to modern language (but see e.g., Di Sciullo, 2013; Miyagawa and Nobrega, 2015 for opposing views). However, these arguments become irrelevant to the problem of evolution of the language capacity once we realize that human language is recursive. Finite language gradually evolving into infinite language is a logical impossibility (section Infinite Cardinal Numbers as Unattainable Limits below). Conceivably, proto-language may have existed as a system of communication (and probably has), but only as an evolutionary *cul-de-sac*, and not as a precursor to human language. Of course, that leaves open the possibility that surface relics and remnants of proto-language may have been incorporated/integrated into the novel and saltationally evolved language phenotype. But this is a different matter altogether and has no relevance for *evolvability* of recursive language. Finally, it is really a speculation outside the reach of empirical testing to argue that constructions like “step-by-step” or (head-initial) exocentric compounds (French *timbre poste, café filtre* and English *scarecrow, killjoy*, or *turncoat*) are “fossils of proto-language.” Language does not fossilize. At best, on current understanding, these cases show analytical problems waiting to be resolved under a unified merge-based approach to syntax and morphology.

## Infinite Cardinal Numbers as Unattainable Limits

P&F confuse the cardinality of (decidable) sets, defined in extension, with the (infinite) productivity of computable functions, defined in intension. A cardinal number is not a specific number *identifying a member* of the set (i.e., an ordinal number, a property of an *individual member*) but rather a number *characterizing the size* of the set (i.e., a property of the *set*). For *finite* sets there is a *greatest* number *n*, the ordinal that has no successor in the set, which can be approximated and reached in a finite number of steps. But for an *infinite* set there is no such greatest number: for any ordinal *n* there is a least ordinal greater than *n* (its *successor* ordinal). Infinite cardinal numbers describe sizes of infinite sets, and may be better thought of as *limit* ordinals. *Lim(n)* = ℵ_0_, where *Aleph-zero* denotes the cardinality of the set of natural numbers, can never be reached by adding up finitely many finite numbers.

Natural language, likewise, is infinite, since there is no longest sentence. Recursive merge may expand a bounded range to an unbounded range of output structures, but no finite set of expressions, however large, can reach unboundedness by combining finitely many finite constructions. A stepwise approach cannot reach infinite generation. Communicative pressures for more signals, necessarily finite numbers of them, could never be pressures for an infinite language. Consequently, the generative procedure couldn't possibly have evolved gradually but must have emerged saltationally without any pressures from the external environment and rather as an exaptive side-effect of cerebral reorganization in *Homo*.

Infinite language has cardinality ℵ_0_. Since cardinality addition and cardinality multiplication do not change cardinality, there is no sense in which infinity can be approximated by a succession of finite additions. But denying precisely that core property lies at the root of P&F's proof of Theorem 2. The proposal is therefore incoherent.

*Theorem 2*. If at least one infinite language has non-zero
  probability (e.g., **P**[ *crd*(*L*) = ∞ ] > 0), then
  **P**[ *crd*(*L_*p*_*) = ∞ | *crd*(*L_*p*_*) > B ] → 1 as B → ∞

The theorem says that “if *L* is constrained to contain at least B strings, *L* will be infinitely productive with increasing probability as B gets large.” Applying the definition of conditional probability P(X|Y) = P(X ∩ Y)/P(Y) to the antecedent of the conditional yields (4).

$$\begin{array}{c} \begin{array}{ll} \left(4\right) & P[crd(L_{p})=\infty |crd(L_{p})>B]=\\ \; & P[crd(L_{p})=\infty ]/(P[crd(L_{p})=\infty ]+P[B<crd(L_{p})<\infty ]) \end{array} \end{array}$$

**Figure 2:**
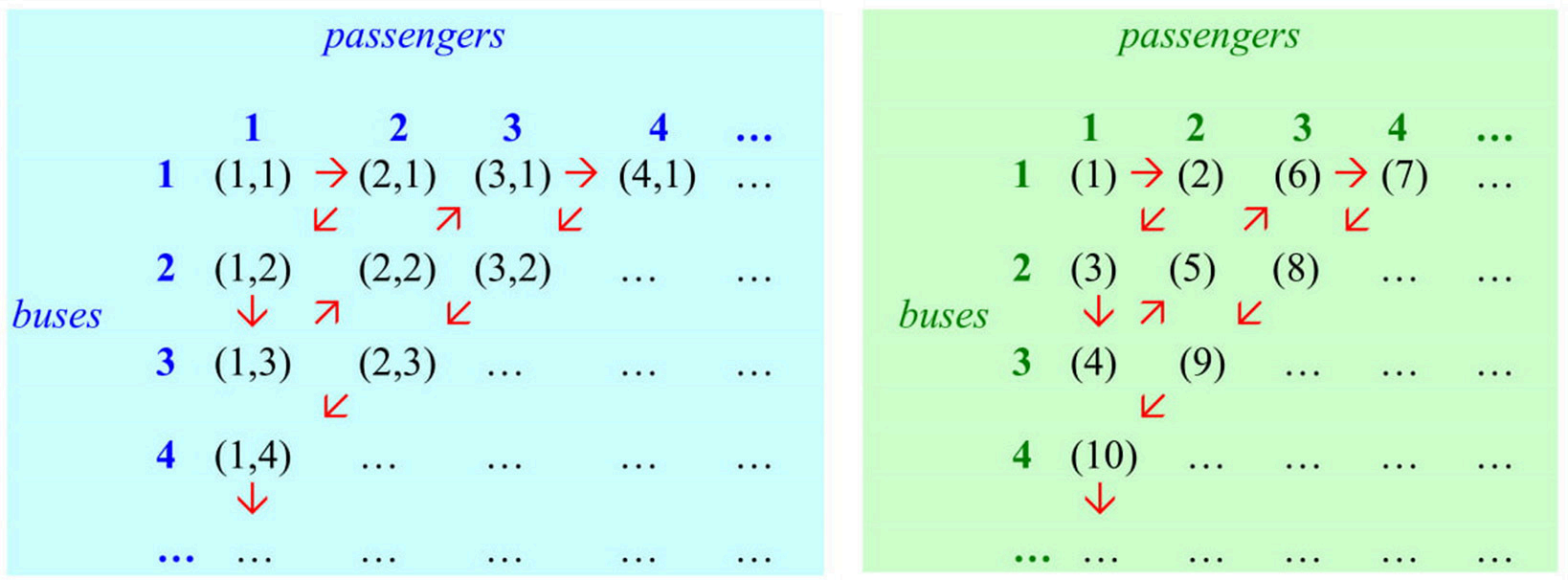
There's always room in Hilbert's Infinite Hotel. Assume that the hotel is already fully booked with an infinite number of permanent guests, and there are arriving an infinite number of coaches, each with an infinite number of seats. Can the new arrivals be suitably accommodated? Yes, they can. *Unvorstellbar* and yet inconceivably true! Strangely enough, the answer is surprisingly simple. For column *X* (passengers) and row *Y* (buses) there is a one-to-one correspondence between the set of ordered pairs of *N* × *N* (representing passenger *x* on bus *y*) and the set of integers *N* (representing room number). *Pairing function:* ***f(x,y)*** = ***[(x***+***y-l)(x***+***y-2)]/2*** **+** ***y***. Since these sets are equivalent, with the same cardinality, *N* × *N* passengers can be accommodated perfectly well in a hotel with *N* rooms (where *N* designates cardinality of the set of positive integers). Diagonal method yields e.g., *(1,1)* **→** *1, (2,1)* **→** *2, (1,2)* **→** *3, (1,3)* **→** *4, (2,2)* **→** *5, (3,1)* **→** *6, (4,1)* **→** *7, …* For example, passenger 3 on bus 1 is given room 6. Here we assume that the permanent guests have arrived at some earlier time with coach 1.

The second term **P**[ B < *crd*(*L*_*p*_) < ∞ ] in the denumerator says that the probability of the size of *L*_*p*_ staying intermediate between cardinality *B* and infinity gets increasingly small as *B* increases. The idea is that the bigger *B* gets, the closer to *Aleph-zero* the size of *L*_*p*_ will be. “As *B* increases, the second term in the denominator must approach zero, meaning that the fraction goes to 1” (P&F, p. 144). However, the probability never reaches zero since it is impossible to reach, or even approximate, infinite generation from finiteness by a stepwise approach adding finitely many finite signals to a finite class of signals. The “quantum jump” from 999,999,999 to infinity is no less than the “jump” from nine to infinity. This is essentially *Hibert's Infinite Hotel* argument (Hilbert, 1924/1926), as explained informally by Gamow (1947) and illustrated in Figure 2.

## Concluding Remarks

The arguments reviewed make (hidden) assumptions that are questionable or even incorrect. Rather than a system of thought, they take language to be basically a means of communication, governed by communicative rather than computational efficiency. They presume that only gradual rather than saltational evolution of a computable function is realistic. Furthermore, they suppose that the study of language concerns decidability of strings (WGC) rather than the *Basic Principle* of language (SGC), and, finally, they ignore the foundational asymmetry of interface mappings, with the mapping to the cognitive systems (internal language) taking primacy over the mapping to the sensorimotor systems (externalization), both in function and evolutionary time. This way, uniformity and universality (principles of UG) are incorrectly dispreferred to diversity, mutability and complexity (parameters and externalization). These and other issues are regrettably ignored or dismissed, but are of paramount importance and scientific interest in studying evolution of language. Here we have argued that unbounded productivity of language is the null hypothesis. The complexity of finite language cannot have been a precursor to the simplicity of infinite generation in evolution of language. Furthermore, no stepwise approach to language can reach infinite generation. Therefore, gradual evolution of recursive language must be ruled out. The effort to make that explicitly clear here will hopefully help safeguard generative grammar against potential charges of contributory negligence.

## Author Contributions

The author confirms being the sole contributor of this work and has approved it for publication.

### Conflict of Interest Statement

The author declares that the research was conducted in the absence of any commercial or financial relationships that could be construed as a potential conflict of interest.
